# Highly Water-Soluble Phenothiazine-Based Quaternary Ammonium Salt Organic Cathode Materials for Organic Flow Batteries

**DOI:** 10.3390/ma19091690

**Published:** 2026-04-22

**Authors:** Guibao Wu, Jianyu Cao, Juan Xu, Mengna Qin, Qun Chen

**Affiliations:** Jiangsu Key Laboratory of Advanced Catalytic Materials and Technology, School of Petrochemical Engineering, Changzhou University, Changzhou 213164, China; 06448123@163.com (G.W.); jycao@cczu.edu.cn (J.C.); cjytion@cczu.edu.cn (J.X.)

**Keywords:** high water solubility, phenothiazine, quaternization, organic flow batteries, cyclic voltammetry

## Abstract

**Highlights:**

- Phenothiazine-based quaternary ammonium salts (PTZQAs) were designed for AORFBs.
- PTZQAs were synthesized using cost-effective raw materials and a facile synthetic route.
- PTZQAs display high solubility in water.
- PTZQAs exhibit a highly positive redox potential and rapid electron transfer kinetics.

**Abstract:**

Organic redox-active molecules are promising catholyte materials for aqueous organic redox flow batteries (AORFBs), yet they often suffer from low solubility and poor cycling stability. Herein, we report a series of water-soluble phenothiazine derivatives functionalized with quaternary ammonium groups. The optimized compound, N,N,N-trimethyl-1-(10H-phenothiazin-10-yl) propan-2-aminium chloride (TMiPrPTCl), exhibits exceptional solubility (2.69 M in water) and a high redox potential (0.902 V vs. SHE). A comparative study of four derivatives reveals that side-chain length and branching critically modulate both solubility and degradation pathways: while three-carbon-linked analogs N,N,N-trimethyl-3-(10H-phenothiazin-10-yl)propan-1-aminium chloride (TMPrPTCl) degrade primarily via irreversible oxidation to sulfoxide, two-carbon-linked species (TMiPrPTCl) undergo additional side-chain cleavage, leading to rapid capacity fade. Although the quaternization strategy successfully achieves record solubility, the electrochemical stability remains a key challenge. Post-cycling analysis confirms the loss of redox activity and the formation of inert products. This work highlights the delicate balance between solubility enhancement and molecular stability, providing clear design guidelines for future phenothiazine-based catholytes.

## 1. Introduction

The rapid deployment of renewable energy sources, especially wind and solar power, has underscored the critical need for scalable energy storage systems to stabilize electrical grids and mitigate intermittency in supply, as extensively documented in the recent literature [1,2]. Among the diverse energy storage systems under development, aqueous redox flow batteries (AORFBs) have gained considerable interest for grid-scale deployment due to their unique benefits, which include decoupled power and energy capacity, long cycle life, high efficiency, inherent safety, and modular scalability [3,4,5]. Currently, the field is dominated by all-vanadium and zinc-bromine systems, which are valued for their high power density and cycling stability [6,7,8]. However, widespread deployment has been hindered by intrinsic limitations: high costs, resource scarcity, toxicity, and the corrosive nature of acidic electrolytes have been associated with vanadium systems [9], while challenges related to bromine volatility, toxicity, and zinc dendrite formation have been observed in zinc–bromine systems [10]. Consequently, research focus has increasingly shifted toward organic-based AORFBs. Composed of earth-abundant elements, organic molecules offer structural diversity and tunability, by which electrochemical properties can be precisely modulated through molecular engineering [11,12]. Despite these promises, the development of organic AORFBs has remained asymmetric. While numerous water-soluble anolyte materials—such as quinones [13,14], viologens [15,16], and TEMPO derivatives [17]—have been successfully deployed, progress in catholyte (positive electrolyte) development has lagged significantly behind. This discrepancy largely stems from the inadequate stability of the highly oxidized, electron-deficient states of organic molecules in aqueous solutions, coupled with the inherent challenge of concurrently attaining high redox potential and substantial solubility [18,19]. Furthermore, the underlying degradation mechanisms of organic catholytes have often been underreported, creating a critical knowledge gap.

Phenothiazine (PTZ) has been identified as a promising class of heterocyclic compounds for catholyte applications due to favorable redox potentials and reversible electrochemistry. Although excellent stability has been demonstrated by phenothiazine derivatives in non-aqueous systems and acidic aqueous systems [20,21,22], their translation to neutral-pH aqueous environments has proven challenging. To address the trade-offs between redox potential, solubility, and kinetics, a strategic molecular design incorporating an electron-withdrawing and hydrophilic quaternary ammonium group into the phenoxazine core is reported herein. Via a facile three-step condensation and quaternization approach, four novel water-soluble catholytes were synthesized, N,N,N-Trimethyl-2-(10H-phenothiazin-10-yl) ethan-1-aminium chloride (TMEtPTCl), N,N,N-trimethyl-3-(10H-phenothiazin-10-yl)propan-1-aminium chloride (TMPrPTCl), N,N,N-trimethyl-1-(10H-phenothiazin-10-yl)propan-2-aminium chloride (TMiPrPTCl) and N,N,N-Trimethyl-3-(2-chloro-10H-phenothiazin-10-yl)propan-1-aminium chloride (TMClPrPTCl), by functionalizing the phenothiazine core with a quaternary ammonium group.

The molecular design and synthesis of a new family of highly water-soluble (>1.6 M) phenothiazine derivatives were performed via quaternization. A systematic comparative structure–property analysis revealing how side-chain length/branching controls redox potential, diffusivity, and, crucially, degradation pathways was also performed. A high redox potential of 0.902 V vs. SHE and exceptional aqueous solubility (~2.69 M) were exhibited by TMiPrPTCl. When paired with a methyl viologen (MV) anolyte, an open-circuit voltage of approximately 1.36 V was delivered by the resulting TMiPrPTCl//MV full cell. Despite these favorable properties, significant capacity decay was revealed by long-term cycling tests. Integrated analysis of battery performance data indicated that TMiPrPTCl underwent irreversible oxidation during electrochemical cycling, by which sulfoxide and sulfone byproducts were yielded. Since these oxidation products were found to be electrochemically inactive, a swift decline in battery capacity was caused by the rapid decomposition of TMiPrPTCl. The demonstration that while high solubility enables promising theoretical energy density, intrinsic chemical instability elucidated through comparative aqueous/non-aqueous cell testing emerges as the primary limitation, providing clear guidance for future molecular engineering.

## 2. Experimental

### 2.1. Materials

Phenothiazine (PT, 98%), Sodium hydride (NaH, 60%), Sodium hydroxide (NaOH, 98%), N,N-Dimethyl-3-bromopropylamine (97%), 1-(dimethylamino)-2-chloropropane hydrochloride (98%), Anhydrous sodium sulfate (Na_2_SO_4_, 99%), Sodium chloride (NaCl, 99.5%), Iodomethane (CH_3_I, 99.5%) and Acetonitrile (MeCN, 99%) were purchased from Aladdin (Shanghai, China). 2-chloro-N,N-dimethylethanamine hydrochloride (98%), Methyl tert-butyl ether (MTBE, 99%), Toluene (99.5%), Acetone (98%), and Hydrochloric acid were purchased from Sinopharm (Shanghai, China). Perphenazine was purchased from Kaiwei (Shanghai, China). Amberlite-IRA-900 anion-exchange resin in the chloride form and methyl viologen (98%) were obtained from Sigma-Aldrich. All experiments were conducted using deionized water (Milli-Q, 18.2 MΩ), and all chemicals were employed as received without further purification.

### 2.2. Synthesis

**Scheme 1:**
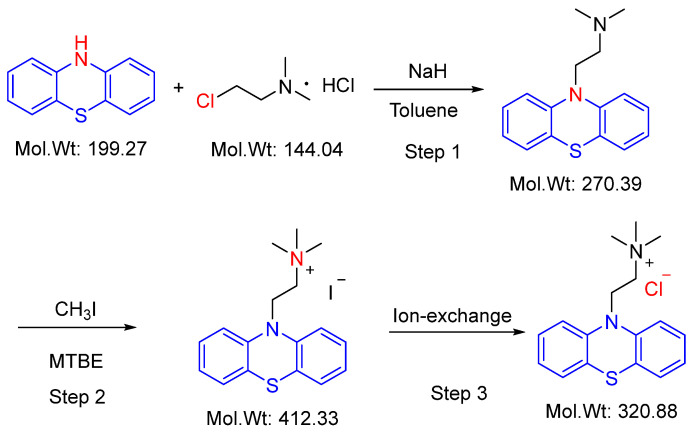
A synthetic route for TMEtPTCl.

Synthesis of N,N,N-Trimethyl-2-(10H-phenothiazin-10-yl)ethan-1-aminium chloride (TMEtPTCl) (Scheme 1): In a 250 mL three-necked round-bottom flask, phenothiazine (5.0 g, 25.09 mmol) and sodium hydride (1.10 g, 27.50 mmol, 60% dispersion in mineral oil) were suspended in toluene (35 mL). The mixture was stirred at 25 °C for 1 h. In a separate beaker, 2-chloro-N,N-dimethylethanamine hydrochloride (7.23 g, 50.19 mmol) was dissolved in water (30 mL). In another container, sodium hydroxide pellets (2.01 g, 50.19 mmol) were dissolved in deionized water (20 mL), and the resulting aqueous NaOH solution was added dropwise to the amine hydrochloride solution. The aqueous layer was then extracted with additional toluene, and the combined toluene extracts, containing the liberated 2-chloro-N,N-dimethylethanamine, were added dropwise via a pressure-equalizing dropping funnel to the phenothiazine suspension. After complete addition, the reaction mixture was stirred at 40 °C for 2 h. The reaction was quenched by adding deionized water (35 mL), and the organic layer was washed sequentially with water and saturated brine. The organic phase was dried over anhydrous Na_2_SO_4_, filtered, and concentrated under reduced pressure. The crude product was purified by column chromatography to afford N,N-dimethyl-2-(10H-phenothiazin-10-yl)ethan-1-amine as a yellow oil (3.73 g, 55.0% yield) [23].

The crude N,N-dimethyl-2-(10H-phenothiazin-10-yl)ethan-1-amine was dissolved in MTBE in a 100 mL three-necked round-bottom flask with stirring. The solution was warmed to 35 °C, and iodomethane (3.92 g, 27.60 mmol) was introduced dropwise using a pressure-equalizing dropping funnel. After maintaining the mixture at this temperature for 8 h, it was cooled to ambient temperature, and the precipitated solid was isolated by filtration. Washing with MTBE and subsequent drying afforded N,N,N-trimethyl-2-(10H-phenothiazin-10-yl)ethan-1-aminium iodide as a pale-yellow powder (5.4 g, 95.0% yield) [24].

An aqueous solution of N,N,N-trimethyl-2-(10H-phenothiazin-10-yl)ethan-1-aminium iodide was slowly passed through a column packed with chloride-form strong-base anion-exchange resin. During the passage, iodide ions were exchanged for chloride ions and retained on the resin matrix. The column was subsequently eluted with deionized water, and the product-containing fractions were collected. The combined aqueous eluates were concentrated under reduced pressure to remove solvent, affording N,N,N-trimethyl-2-(10H-phenothiazin-10-yl)ethan-1-aminium chloride as a dark yellow solid (3.73 g, 95.8% yield). ^1^H NMR (500 MHz, DMSO-d_6_): *δ* 7.25–7.35(m, 6H, Ar H), 7.08(t, *J* = 10, 2H, Ar H), 4.44(t, *J* = 10, 2H, -CH_2_-), 3.66(t, *J* = 7.5, 2H, -CH_2_-), 3.18(s, 9H, -CH_3_).

**Scheme 2:**
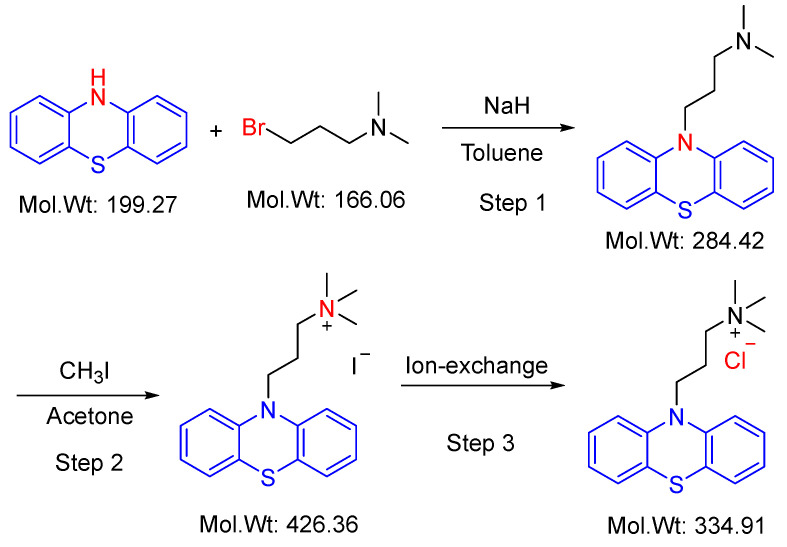
A synthetic route for TMPrPTCl.

Synthesis of N,N,N-trimethyl-3-(10H-phenothiazin-10-yl)propan-1-aminium chloride (TMPrPTCl) (Scheme 2): TMPrPTCl was obtained by using a similar synthetic route with N,N-Dimethyl-3-bromopropylamine as an initial reactant. ^1^H NMR (500 MHz, DMSO-d_6_): *δ* 7.21–7.29(m, 4H, Ar H), 7.12(d, *J* = 10, 2H, Ar H), 7.01(t, *J* = 10, 2H, Ar H), 3.97(t, *J* = 7.5, 2H, -CH_2_-), 3.43(m, 2H, -CH_2_-), 3.04(s, 9H, -CH_3_), 2.13(m, 2H, -CH_2_-).

**Scheme 3:**
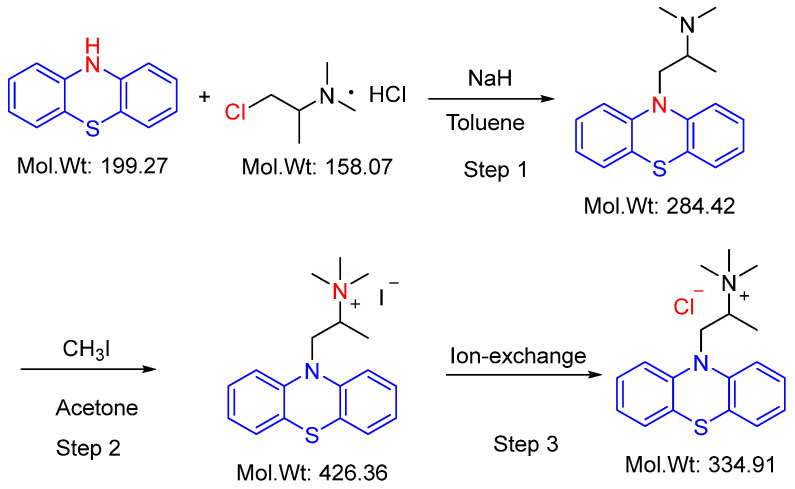
A synthetic route for TMiPrPTCl.

Synthesis of N,N,N-trimethyl-1-(10H-phenothiazin-10-yl)propan-2-aminium chloride (TMiPrPTCl) (Scheme 3): TMiPrPTCl was obtained by using a similar synthetic route with 1-(dimethylamino)-2-chloropropane hydrochloride as an initial reactant. ^1^H NMR (500 MHz, DMSO-d_6_): *δ* 7.23–7.33(m, 6H, Ar H), 7.06(t, *J* = 10, 2H, Ar H), 4.64(dd, *J*_1_ = 5, *J*_2_ = 20, 1H, -CH_2_-), 4.06(m, 1H, -CH_2_-), 3.75(m, H, -CH-), 3.15(s, 9H, -CH_3_), 1.38(d, *J* = 5, 3H, -CH_3_).

**Scheme 4:**
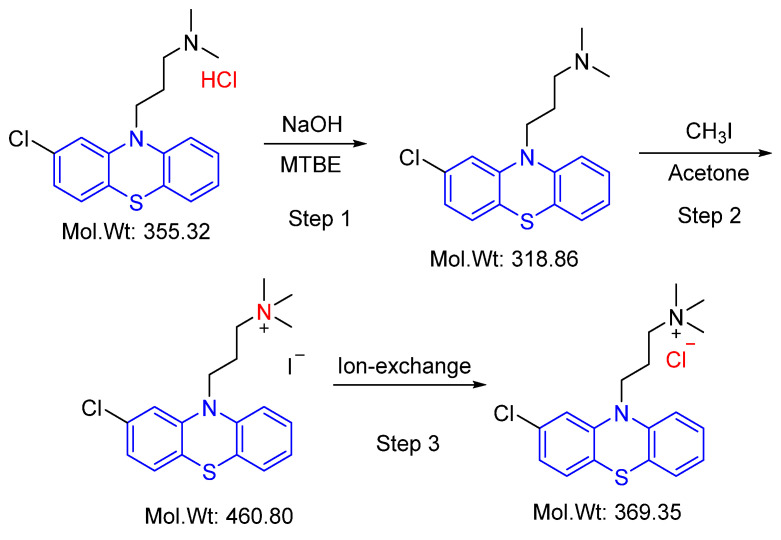
A synthetic route for TMClPrPTCl.

Synthesis of N,N,N-Trimethyl-3-(2-chloro-10H-phenothiazin-10-yl)propan-1-aminium chloride (TMClPrPTCl) (Scheme 4): TMClPrPTCl was obtained by using a similar synthetic route with chlorpromazine hydrochloride as an initial reactant. ^1^H NMR (500 MHz, DMSO-d_6_): *δ* 7.21–7.29(m, 3H, Ar H), 7.17(d, *J* = 5, 1H, Ar H), 7.12(d, *J* = 10, 1H, Ar H), 7.04(m, 2H, Ar H), 3.97(t, *J* = 7.5, 2H, -CH_2_-), 3.41(m, 2H, -CH_2_-), 3.03(s, 9H, -CH_3_), 2.10(m, 2H, -CH_2_-).

### 2.3. Characterization

Proton nuclear magnetic resonance (^1^H NMR) spectra were acquired on a Bruker ARX-500 spectrometer using deuterated dimethyl sulfoxide (DMSO-d_6_) as the solvent. Solubility measurements of TMEtPTCl, TMPrPTCl, TMiPrPTCl and TMClPrPTCl were performed at room temperature with a Shimadzu UV mini 1240 UV–vis spectrophotometer. Fourier transform infrared (FT-IR) spectra were recorded on a Thermo Scientific Nicolet Nexus 670 spectrometer over the wavenumber range of 675–4000 cm^−1^.

### 2.4. Electrochemical Measurements

Electrochemical measurements were carried out on a CHI 660B workstation (CH Instruments) in a nitrogen-purged glovebox maintained at 24 ± 1°C. A conventional three-electrode setup was used, consisting of a platinum wire counter electrode and a saturated calomel electrode (SCE) as the reference, with 1 M NaCl aqueous solution as the electrolyte. Prior to testing, dissolved oxygen was removed by purging the solutions with high-purity nitrogen for 20 min. Cyclic voltammetry (CV) was performed at multiple scan rates. All potentials measured versus SCE were converted to the standard hydrogen electrode (SHE) scale by adding 0.242 V.

Linear sweep voltammetry (LSV) was performed to determine the diffusion coefficient (D) using a CHI 600B potentiostat integrated (CH Instruments, Bee Cave, TX, USA) with an ALS RRDE-3A rotating disk electrode system. A three-electrode configuration was employed: a glassy carbon rotating disk as the working electrode, a platinum wire counter electrode, and a saturated calomel electrode (SCE) reference. The LSV curves were recorded at a constant scan rate of 10 mV s^−1^ while varying the rotation rate from 200 to 2500 rpm. D was derived from the slope of the Levich plot according to the Levich equation: *i* = 0.620*nFAC*_0_*D*^2/3^*ω*^1/2^*υ*^−1/6^, where *n* (taken as 1) is the number of electrons transferred, *F* is Faraday’s constant (96,485 C mol^−1^), *A* is the geometric area of the electrode (0.1963 cm^2^), C_0_ is the bulk concentration of the redox species (1 × 10^−6^ mol cm^−3^), *ω* is the angular velocity (rad s^−1^), and *υ* is the kinematic viscosity (approximated as 0.01 cm^2^ s^−1^ for 1 M NaCl solution).

### 2.5. Flow Cell Measurements

The flow cell was assembled with commercially available components sourced from Fuel Cell Technologies Inc. (Albuquerque, NM, USA). The assembly included metallic end plates, gold-plated current collectors, and serpentine-channel graphite flow field plates. This configuration provided an active geometric area of 5 cm^2^. Electrochemical performance was evaluated using a Neware battery testing system. To isolate the anolyte and catholyte compartments, a 100 μm thick AMV anion-exchange membrane (Asahi Glass, Tokyo, Japan) was installed. The electrode stack consisted of acid-treated hydrophilic graphite felt (2 mm thickness, CeTech, Taichung City, Taiwan), following the protocol described in Ref. [25]. The assembly conditions for aqueous flow battery systems were as follows: the solvent was water, the positive electrolyte (catholyte) comprised 10 mL of 0.1 M TMiPrPTCl dissolved in 1 M NaCl, and the negative electrolyte (anolyte) consisted of 10 mL of 0.1 M MV in 1 M NaCl. The assembly conditions for non-aqueous flow battery systems were as follows: the solvent was MeCN, the positive electrolyte (catholyte) comprised 10 mL of 0.1 M TMiPrPTCl dissolved in 1 M TBAPF_6_, and the negative electrolyte (anolyte) consisted of 10 mL of 0.1 M MV in 1 M TBAPF_6_. Both electrolytes were circulated at a constant rate of 30 mL min^−1^ utilizing BT-600EA peristaltic pumps (Chongqing Jieheng, Chongqing, China).

## 3. Results and Discussion

The target compound, TMiPrPTCl (N,N,N-trimethyl-1-(10H-phenothiazin-10-yl) propan-2-aminium chloride), was synthesized via a three-step protocol involving the N-alkylation of phenothiazine with 1-(dimethylamino)-2-chloropropane hydrochloride, followed by quaternization and anion exchange (Scheme 3). The purity and redox-active characteristics of the product were verified by ^1^H NMR spectroscopy (Figures S1–S3). To elucidate the influence of linker length and substituents on electrochemical performance, a series of structurally related phenothiazine derivatives—specifically TMEtPTCl (ethyl linker), TMPrPTCl (propyl linker), and TMClPrPTCl (2-chloro substituted)—were prepared utilizing the same synthetic strategy (Scheme 1, Scheme 2 and Scheme 4). The chemical structures of all synthesized compounds were fully characterized and confirmed through ^1^H NMR and FT-IR analysis (Figures S1 and S2).

**Figure 1:**
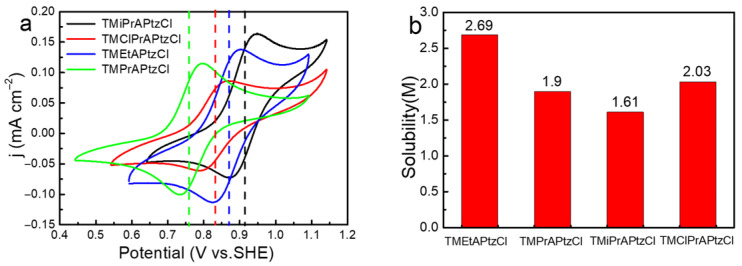
(**a**) CVs of 1 mM TMEtPTCl (blue line), TMPrPTCl (green line), TMiPrPTCl (black line), TMClPrPTCl (red line) in 1 M NaCl solution at scan rates of 25 mV s^−1^. (**b**) Solubilities of TMEtPTCl, TMPrPTCl, TMiPrPTCl and TMClPrPTCl in water at room temperature.

To address the poor aqueous solubility inherent to unsubstituted phenothiazine, we introduced hydrophilic quaternary ammonium functionalities into the molecular structure. This strategy proved highly effective: the derivatives TMEtPTCl, TMPrPTCl, TMiPrPTCl and TMClPrPTCl display exceptional water solubilities of 1.6 M, 2.03 M, 2.69 M and 1.9 M, respectively (Figure 1b). These values significantly exceed those of the parent compound, highlighting the potential of these quaternized species for use in high-performance AORFBs.

**Figure 2:**
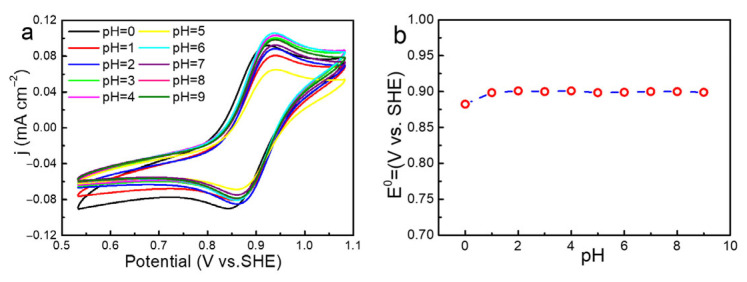
(**a**) CVs of 1 mM TMiPrPTCl in unbuffered NaCl solution (1 M) with different pHs (0–9). (**b**) Pourbaix diagram (E^0^ vs pH) for TMiPrPTCl.

Excellent electrochemical reversibility in 1 M NaCl was demonstrated by TMEtPTCl, TMPrPTCl, TMiPrPTCl, and TMClPrPTCl, as revealed in Figure 1a and Table 1, with Δ*Ep* values of approximately 77, 61, 73, and 86 mV, respectively. A distinct variation in redox potential was observed: a higher operating potential was exhibited by TMiPrPTCl (E^0^ = 0.902 V vs. SHE) compared to TMEtPTCl (E^0^ = 0.860 V vs. SHE), TMPrPTCl (E^0^ = 0.754 V vs. SHE) and TMClPrPTCl (E^0^ = 0.832 V vs. SHE). The diffusion-controlled nature of the reaction was validated by the linear increase in peak current density against *v*^1/2^ (inset, Figure S3). Furthermore, within the pH range of 0–9, the redox potential of TMiPrPTCl was found to be insensitive to pH variations, as indicated by pH-dependent studies conducted in unbuffered 1 M NaCl (Figure 2).

**Figure 3:**
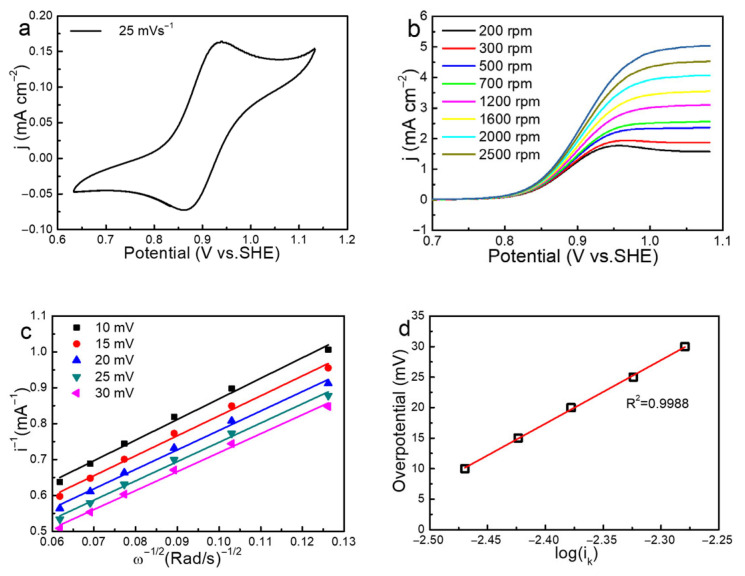
(**a**) CV curves of 1 mM TMiPrPTCl in 1 M NaCl solution at a scan rate of 25 mV s^−1^. (**b**) RDE voltammetry curves of 1 mM TMiPrPTCl in 1 M NaCl solution at 10 rotation rates ranging from 200 to 2500 rpm. (**c**) Levich plot of limiting current versus square root of rotation rate (ω^−1/2^). (**d**) Tafel plots for 1 mM TMiPrPTCl in 1 M NaCl solution on a bare glassy carbon electrode.

The diffusion coefficients (D) of TMEtPTCl, TMPrPTCl, TMiPrPTCl, and TMClPrPTCl were determined via rotating disk electrode (RDE) voltammetry (Figure 3) to be 1.51 × 10^−5^ cm^2^ s^−1^, 6.71 × 10^−6^ cm^2^ s^−1^, 2.29 × 10^−6^ cm^2^ s^−1^ and 2.72 × 10^−6^ cm^2^ s^−1^, respectively. Faster reaction kinetics were exhibited by TMEtPTCl compared to the other derivatives (Table 1). Mass and charge transfer resistances are effectively reduced by the combination of high diffusivity and rapid electron transfer kinetics, thereby leading to enhanced energy efficiency in redox flow batteries (RFBs) [26]. Although relatively sluggish kinetics were observed for TMiPrPTCl in comparison to the other candidates, it was selected for full-cell assembly due to its exceptionally high solubility, which exceeds that of the other three compounds. It is indicated by theoretical analysis that volumetric energy density in flow battery systems is positively correlated with the solubility of active species; consequently, the enhancement of solubility is regarded as a critical strategy for maximizing the theoretical energy storage capacity per unit volume [27,28,29].

**Figure 4:**
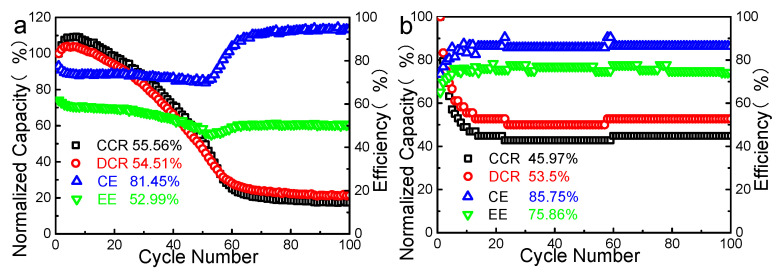
TMiPrPTCl//MV cell: Catholyte: 10 mL of 0.1 M TMiPrPTCl; Anolyte: 10 mL of 0.1 M MV. The normalized capacity is evaluated on the basis of the capacity of the 1st charge–discharge cycle. (**a**) Solvent: H_2_O, Supporting Electrolyte: 1 M NaCl; (**b**) Solvent: MeCN, Supporting Electrolyte: 1 M TBAPF_6_.

A neutral-pH aqueous all-organic redox flow battery (RFB) was fabricated leveraging the enhanced properties of TMiPrPTCl to validate performance. The cell configuration comprised 10 mL of 0.1 M TMiPrPTCl catholyte and 20 mL of 0.1 M methyl viologen (MV) anolyte in a 1 M NaCl supporting electrolyte (Figure 4a). A potential cell voltage of 1.36 V was suggested by separate cyclic voltammetry (CV) scans. The long-term cycling performance of the TMiPrPTCl//MV cell was evaluated at a current density of 5 mA cm^−2^. The evolution of discharge capacity retention (DCR, red circles), coulombic efficiency (CE, blue triangles), and energy efficiency (EE, green inverted triangles) was plotted against the cycle number. After 100 cycles, average values of 54.51% (DCR), 81.45% (CE), and 52.89% (EE) were recorded. During the initial cycling regime, a precipitous decline in DCR from near-unity values was exhibited, indicative of substantial irreversible capacity loss occurring during the activation phase or early-stage cycling. The observed average CE of 81.45% was found to be markedly inferior to benchmarks established for mature electrochemical energy storage systems, wherein lithium-ion batteries typically exhibit CE > 99% and high-performance RFBs exceed 95% [30,31]. This substantial deviation strongly implies the prevalence of persistent parasitic side reactions throughout the charge/discharge processes. In organic-based systems, such low CE is likely attributed to electrolyte decomposition or the irreversible oxidation/reduction of active species [32].

To mitigate the rapid degradation observed in aqueous electrolytes, a non-aqueous redox flow battery (NAORFB) employing acetonitrile as the solvent was assembled. Except for the use of tetrabutylammonium hexafluorophosphate (TBAPF_6_) as the supporting electrolyte and MeCN as the solvent for both the positive and negative electrodes, all other components of the electrode systems, including the active materials, their concentrations, electrolyte volumes, separators and electrode materials, remained identical to those used in the aqueous configuration (Figure 4b). A significant divergence between the average coulombic efficiency (CE, 85.75%) and energy efficiency (EE, 75.86%) was highlighted, underscoring the substantial voltage losses inherent to non-aqueous electrolytes, which are primarily driven by high ohmic resistance and sluggish reaction kinetics [33,34]. However, the most critical observation was the catastrophic decay of charge capacity (CCR) and discharge capacity (DCR), which emphasized the fragility of active species in organic solvents despite moderate efficiency retention. The rapid capacity attenuation evident in the DCR curve was indicative of irreversible active material loss, a pervasive challenge in NAORFBs. Although a CE of ~86% suggested reasonable charge reversibility on a per-cycle basis, the cumulative impact of ~14% inefficiency per cycle was found to drive the precipitous capacity drop. The discrepancy between CE and EE implied that a portion of the input energy was consumed by parasitic chemical reactions rather than being stored chemically. The sharp decline in capacity suggested that the active species (or the supporting electrolyte) underwent irreversible decomposition—such as radical dimerization, nucleophilic attack by solvent molecules, or instability at extreme potentials—resulting in a permanent loss of electroactive concentration [34,35]. The observation that EE remained relatively stable around 75% while capacity collapsed suggested that the kinetics of the remaining active species were preserved, whereas the quantity of the active inventory was rapidly depleted. This decoupling confirmed that the failure mode was attributed to the stoichiometric exhaustion of the active material via side reactions [36].

**Figure 5:**
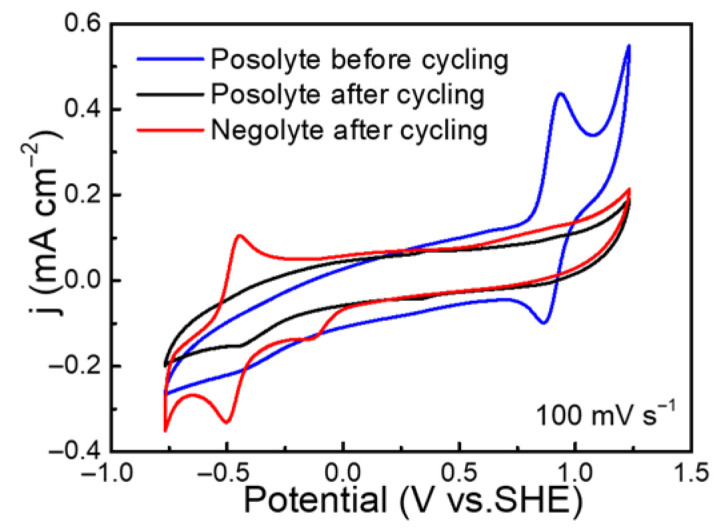
CVs of the 1 mM TMiPrPTCl posolyte before and after cycling and the 1 mM MV negolyte after cycling obtained at 100 mVs^−1^ on the GC electrode.

Based on the results shown in Figure 4, rapid capacity fading of the active material TMiPrPTCl is observed in both aqueous and non-aqueous systems. The most direct performance indicator is the sharp decline in charge and discharge capacities over a relatively small number of cycles. This decay does not represent the gradual loss typically associated with mechanical crossover or minor side reactions but rather a steep attenuation, indicating a rapid and continuous depletion of the active material concentration. The failure of subsequent charging steps to restore the initial capacity confirms that the loss is chemically irreversible; the active material is not merely confined in an inactive form but is converted into electrochemically inert products. The persistently low coulombic efficiency (averaging approximately 81.5% in aqueous and 85.8% in non-aqueous systems) serves as a quantitative signature of parasitic side reactions. A coulombic efficiency below 100% indicates that not all charge passed during charging is recovered during discharge. This lost charge is directly consumed by chemical side reactions. The continuous presence of low coulombic efficiency throughout cycling demonstrates that these are not one-time activation processes but ongoing parasitic pathways that compete with the target redox reaction. After cycling in the aqueous system, the catholyte and anolyte solutions were diluted to a concentration of 1 mM for cyclic voltammetry (CV) testing, and the results were compared with those of the fresh catholyte at the same concentration (Figure 5). Changes in the CV profiles before and after cycling provide molecular-level insights. The disappearance of the redox peaks in the cycled catholyte indicates that new chemical species are generated through side reactions, and these newly formed species lack redox activity. In summary, the electrochemical data, combining performance metrics (capacity, coulombic efficiency, and energy efficiency) with the evolution of CV profiles, depict a scenario inconsistent with a simple, reversible redox process. Instead, it is fully consistent with a system undergoing severe chemical side reactions leading to the irreversible decomposition of the active material.

**Figure 6:**
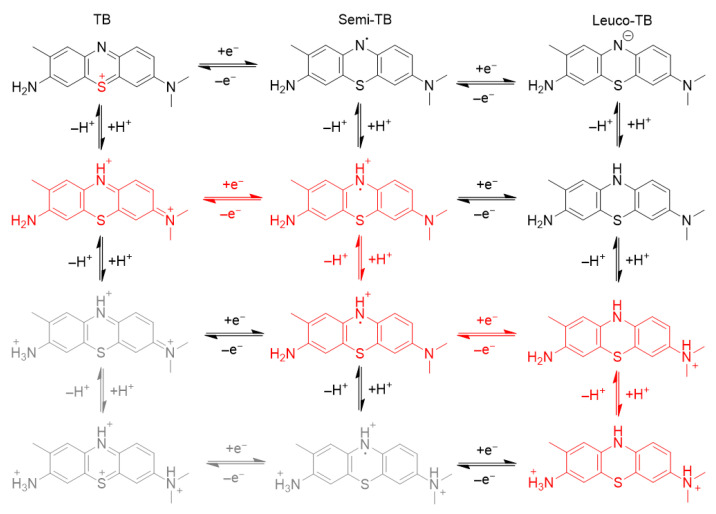
The transformation process of the toluidine blue (TB)/leucomethylene blue (Leuco-TB) redox reaction. (The gray molecules are not present; the red molecules represent the redox process of toluidine blue (TB) in 3 M sulfuric acid (H_2_SO_4_)).

**Figure 7:**
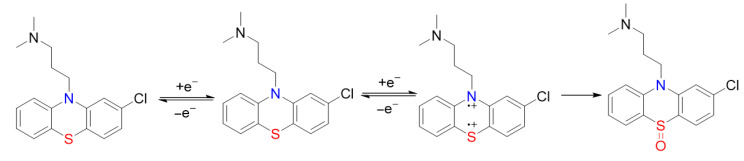
Mechanism of the Redox Behavior of Chlorpromazine.

The solubility and redox potential of the synthesized materials exhibit significant advantages compared to previously reported phenothiazine dye derivatives, such as methylene blue (MB) [21] and 3,7-bis[(2-hydroxyethyl)(methyl)amino]phenothiazin-5-ium bromide (BHAP) [22]. However, their stability remains considerably inferior. The redox mechanisms of the two types of phenothiazine derivatives, phenothiazine dye derivatives and N-alkyl-substituted phenothiazine derivatives, differ significantly. In phenothiazine dye derivatives, the tricyclic structure contains a sulfonium cation, which forms a salt with a chloride or acetate anion. Their redox reactions involve a two-electron transfer at the nitrogen atom and the aromatic ring, as illustrated in Figure 6 [37]. In contrast, N-substituted phenothiazines (N-PTZs), such as chlorpromazine, can donate one electron at the nitrogen site to form a radical cation. However, the donation of a second electron leads to significant molecular instability, resulting in irreversible oxidation to the corresponding sulfoxide, as shown in Figure 7 [38].

**Figure 8:**
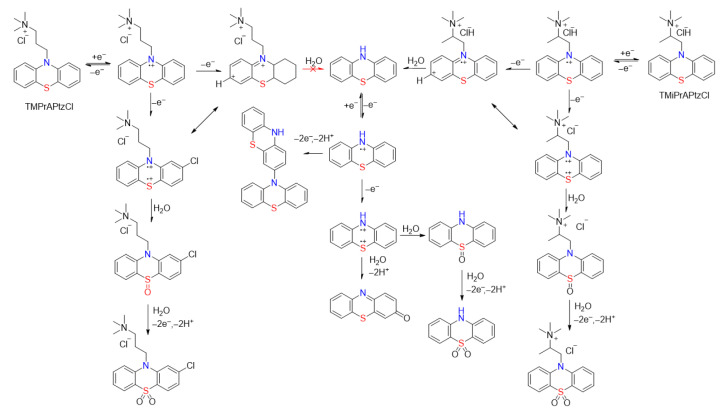
Electrochemical Degradation Pathways of TMPrPTCl and TMiPrPTCl.

J.-M. Kauffmann et al. investigated the oxidation of several phenothiazine drugs, including phenothiazine, promethazine hydrochloride, promazine hydrochloride, trimeprazine hydrochloride, and ethopropazine hydrochloride, in an acidic aqueous medium using electrochemical, chemical, and enzymatic methods. Molecules with two carbon atoms (2C) separating the ring nitrogen from the terminal nitrogen in the side chain exhibited two parallel oxidation pathways: (i) formation of the corresponding sulfoxide and (ii) cleavage of the side chain with release of phenothiazine oxidation products (phenothiazine sulfoxide and phenothiazine quinone imine). In contrast, molecules with three intervening carbon atoms (3C) between the two nitrogen atoms underwent oxidation solely to the corresponding sulfoxide. The difference in oxidation behavior between 2C and 3C molecules may be attributed to steric effects arising from the side chain structure (Figure 8).

The synthesized N-alkyl-substituted phenothiazine derivatives can be categorized into two groups. The first group consists of TMPrAPtzCl and TMClPrAPtzCl, in which three carbon atoms (3C) separate the two nitrogen atoms; TMPrAPtzCl is used as an example in the figure. The second group includes TMEtAPtzCl and TMiPrAPtzCl, where two carbon atoms (2C) separate the ring nitrogen from the terminal nitrogen; TMiPrAPtzCl is used as an example in the figure. The degradation mechanisms of the two groups are illustrated in Figure 8. In the first group, TMPrAPtzCl loses one electron to form a radical cation, a process that is electrochemically reversible. However, the loss of a second electron leads to significant molecular instability, followed by reaction with water to yield the corresponding sulfoxide. At this stage, TMPrAPtzCl loses its reversible redox activity, though the sulfoxide can undergo further oxidation to form the sulfone. Because TMPrAPtzCl and TMClPrAPtzCl possess a 3C spacer between the two nitrogen atoms, the side chain is not easily cleaved, and sulfoxide/sulfone formation represents the sole degradation pathway for these compounds. In contrast, molecules of the second group, exemplified by TMiPrAPtzCl, also undergo irreversible oxidation to the corresponding sulfoxide and sulfone. Additionally, TMiPrAPtzCl can undergo elimination of the quaternary ammonium side chain via resonance stabilization of the radical cation. Once the side chain is lost, the molecule reverts to the phenothiazine core, which subsequently precipitates from the electrolyte. As noted in the previous literature, the phenothiazine core may then undergo disproportionation, dimerization, irreversible oxidation, and other side reactions. Therefore, TMEtAPtzCl and TMiPrAPtzCl all degrade electrochemically via these two parallel pathways.

## 4. Conclusions

In conclusion, the strategic integration of a quaternary ammonium group into the redox-active phenothiazine core yielded PTZQAs, four novel organic catholytes with exceptional water solubility for aqueous organic redox flow batteries (AORFBs). TMiPrPTCl demonstrates a high redox potential of 0.902 V vs. SHE, and the diffusion coefficient (D) was determined to be 2.29 × 10^−6^ cm^2^ s^−1^ in neutral pH electrolytes. Despite its highly reversible electrochemical behavior, the material suffers from rapid capacity fading within tens of cycles. Integrated analysis of battery performance data indicates that TMiPrPTCl undergoes irreversible oxidation during electrochemical cycling, yielding sulfoxide and sulfone byproducts. Since these oxidation products are electrochemically inactive, the rapid decomposition of TMiPrPTCl leads to a swift decline in battery capacity.

## 5. Patents

The author Wu Guibao is the inventor of a Chinese Patent Application CN 202411557291.X titled Cathodic Organic Electrolytes with Phenothiazine Quaternary Ammonium Salt Structures, Their Preparation Methods, and Applications, which covers [Phenothiazine Quaternary Ammonium Salts preparation methods]. The patent was filed on 15 November 2024 and published 21 February 2025 by Changzhou University.

## Figures and Tables

**Table 1:** A summary of physicochemical/electrochemical properties of phenothiazine quaternary ammonium (PTZQA) salts.

Compound	[C]in H_2_O(M)	E^0^ (vs. SHE)	ΔE (mV)	D × 10^5^ (cm^2^ s^−1^)
TMEtPTCl	>1.61	0.860	77	1.51 × 10^−5^
TMPrPTCl	2.03	0.754	61	6.71 × 10^−6^
TMiPrPTCl	2.69	0.902	73	2.29 × 10^−6^
TMClPrPTCl	1.90	0.832	86	2.72 × 10^−6^

## Data Availability

The original contributions presented in this study are included in the article/Supplementary Material. Further inquiries can be directed to the corresponding authors.

## Supplementary Materials

The following supporting information can be downloaded at: https://www.mdpi.com/article/10.3390/ma19091690/s1, Figure S1: ^1^H NMR Spectrum of TMEtAPtzCl, TMPrAPtzCl, TMiPrAPtzCl, TMClPrAPtzCl; Figure S2: FT-IR of TMEtAPtzCl, TMPrAPtzCl, TMiPrAPtzCl, TMClPrAPtzCl; Figure S3: (a–d) CVs of 1 mM TMEtAPtzCl (a), TMPrAPtzCl (b), TMiPrAPtzCl (c) and TMClPrAPtzCl (d) in 1 M NaCl solution at scan rates of 5~200 mV s^−1^; Figure S4-1: (a) UV-Vis absorption spectra of TMEtAPtzCl solutions with varying concentrations in deionized water. (b) Linear plot of TMEtAPtzCl concentration against the maximum absorbance recorded at λ = 250 nm (10,000-fold dilution). The solubility of TMEtAPtzCl in deionized water was determined to ~>1.61 M at 25 ± 1 °C (calculated based on 1 L of solvent); Figure S4-2: (a) UV-Vis absorption spectra of TMPrAPtzCl solutions with varying concentrations in deionized water. (b) Linear plot of TMPrAPtzCl concentration against the maximum absorbance recorded at λ = 252 nm (10,000-fold dilution). The solubility of TMPrAPtzCl in deionized water was determined to be 2.03 M at 25 ± 1 °C (calculated based on 1 L of solvent); Figure S4-3: (a) UV-Vis absorption spectra of TMiPrAPtzCl solutions with varying concentrations in deionized water. (b) Linear plot of TMiPrAPtzCl concentration against the maximum absorbance recorded at λ = 251 nm (10,000-fold dilution). The solubility of TMiPrAPtzCl in deionized water was determined to be 2.69 M at 25 ± 1 °C (calculated based on 1 L of solvent); Figure S4-4: (a) UV-Vis absorption spectra of TMClPrAPtzCl solutions with varying concentrations in deionized water. (b) Linear plot of TMClPrAPtzCl concentration against the maximum absorbance recorded at λ = 310 nm (10,000-fold dilution). The solubility of TMClPrAPtzCl in deionized water was determined to be 1.90 M at 25 ± 1 °C (calculated based on 1 L of solvent).
