# Supplementary material for: Highly Water-Soluble Phenothiazine-Based Quaternary Ammonium Salt Organic Cathode Materials for Organic Flow Batteries
Source: Materials (Basel). 2026 Apr 22;19(9):1690. doi: 10.3390/ma19091690 (PMC13164639; doi:10.3390/ma19091690)
Supplement: Supplementary file 1 [file materials-19-01690-s001.zip › materials-4239603-supplementary.pdf]

Supporting Information

High water-soluble Phenothiazine-based quaternary ammonium salts organic cathode materials for organic flow batteries

Guibao Wu, Jianyu Cao, Juan Xu, Qun Chen* and Mengna Qin*

Jiangsu Key Laboratory of Advanced Catalytic Materials and Technology, School of Petrochemical Engineering, Changzhou University, Changzhou 213164, China

*Corresponding author,

Tel. & Fax: +86-0519-86330239

E-mail: mengnaqin080516@163.com; chenqun@cczu.edu.cn

Figure S1

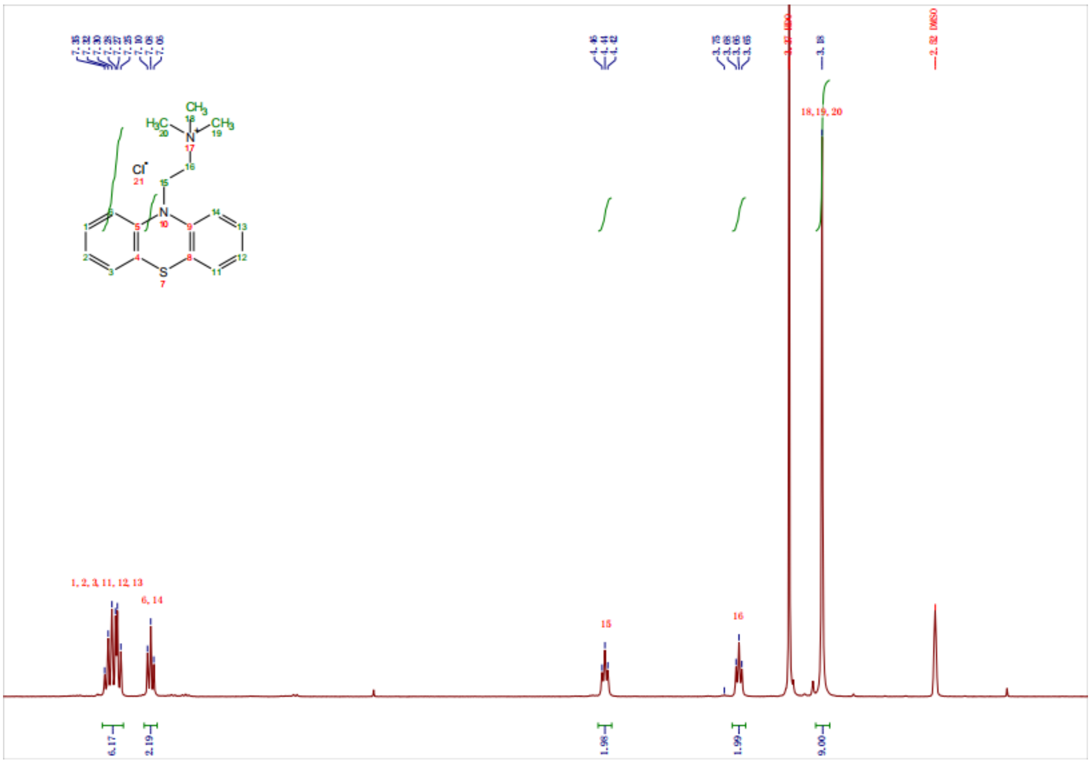

Figure S1-1 ^{1}H NMR Spectrum of TMEtAPtzCl.

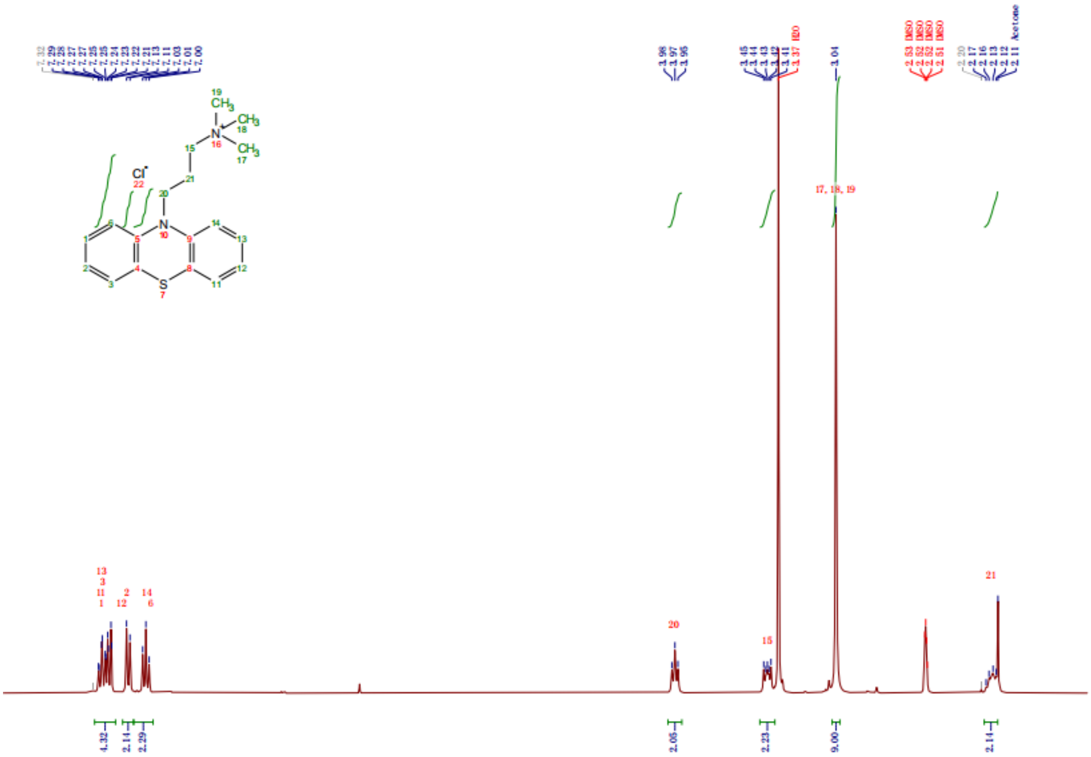

Figure S1-2 ^{1}H NMR Spectrum of TMPrAPtzCl.

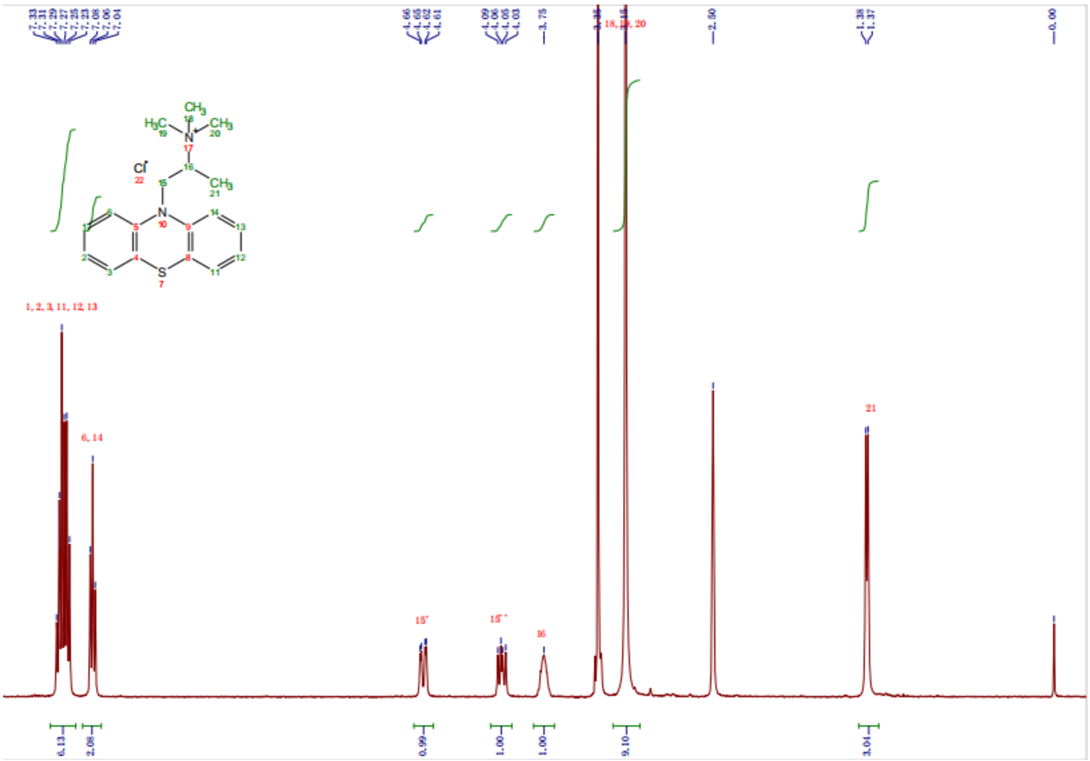

Figure S1-3 ^{1}H NMR Spectrum of TMiPrAPtzCl.

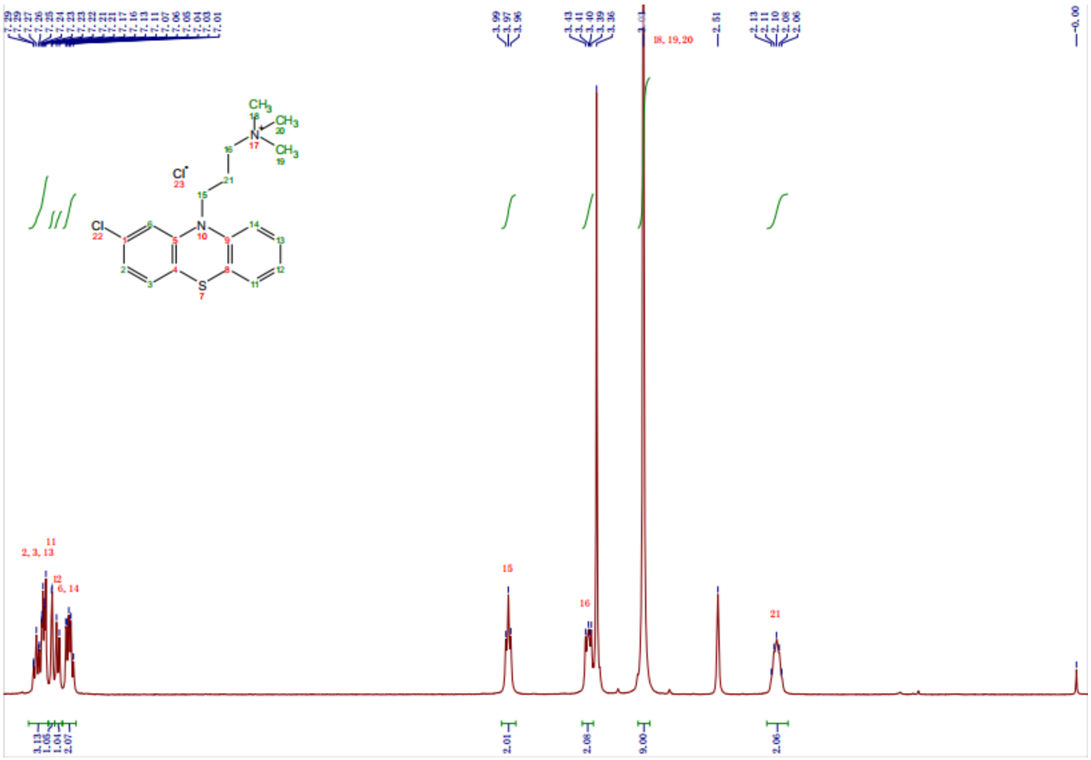

Figure S1-4 ^{1}H NMR Spectrum of TMClPrAPtzCl.

Figure S2

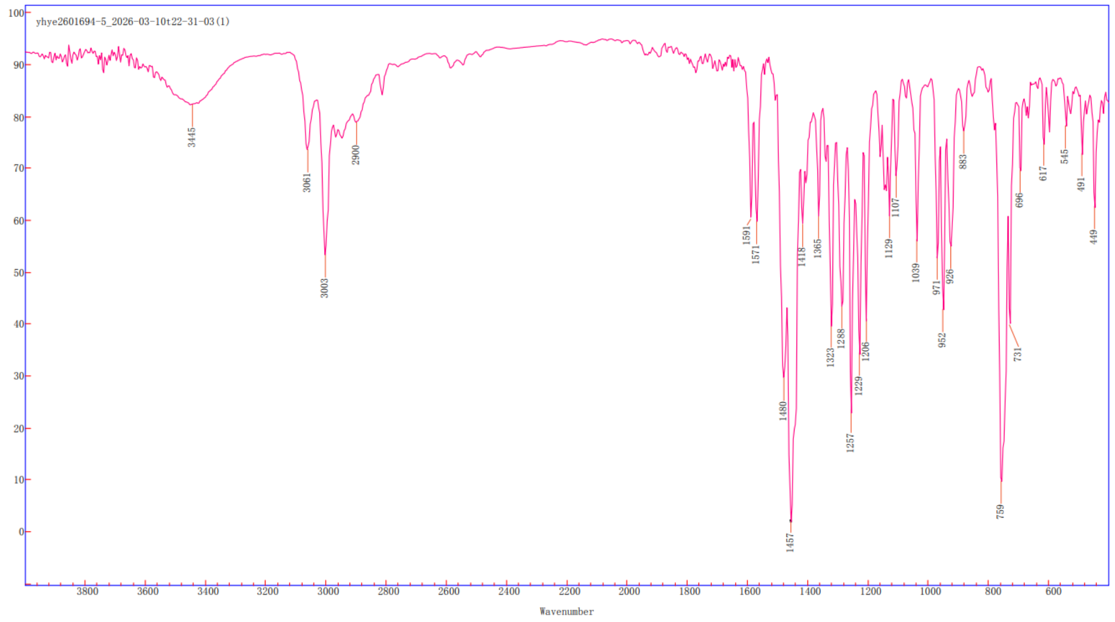

Figure S2-1 FT-IR of TMEtAPtzCl.

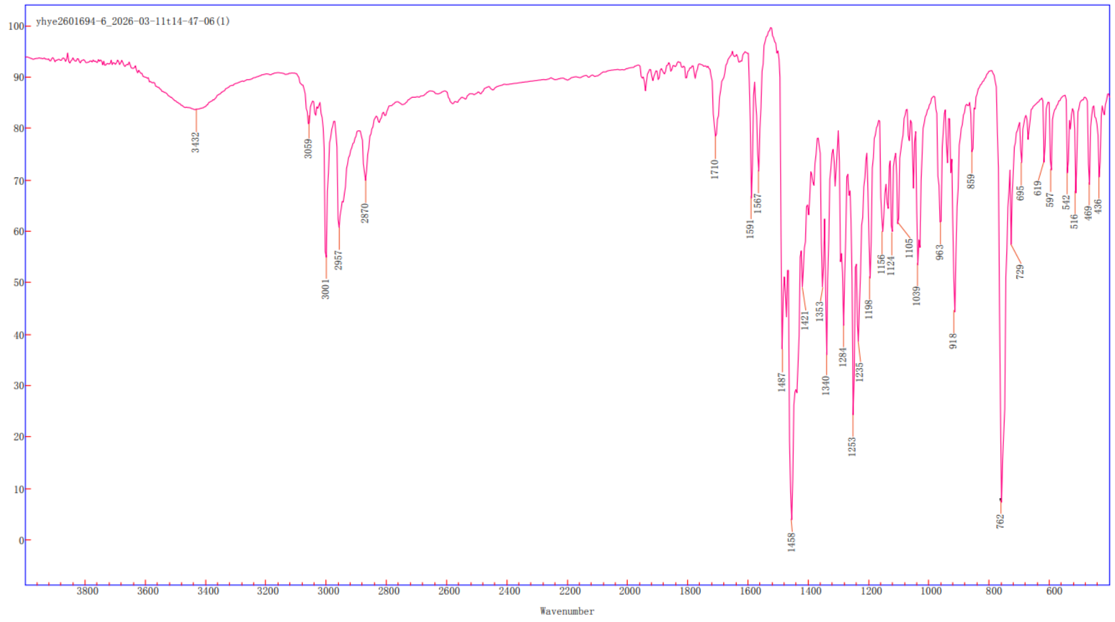

Figure S2-2 FT-IR of TMPrAPtzCl.

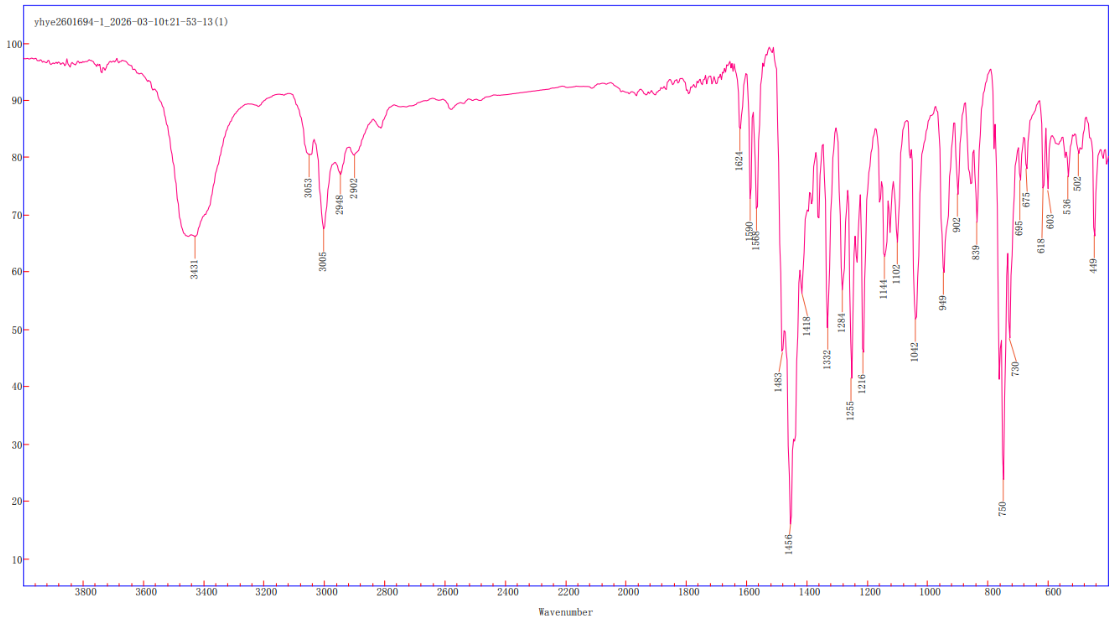

Figure S2-3 FT-IR of TMiPrAPtzCl.

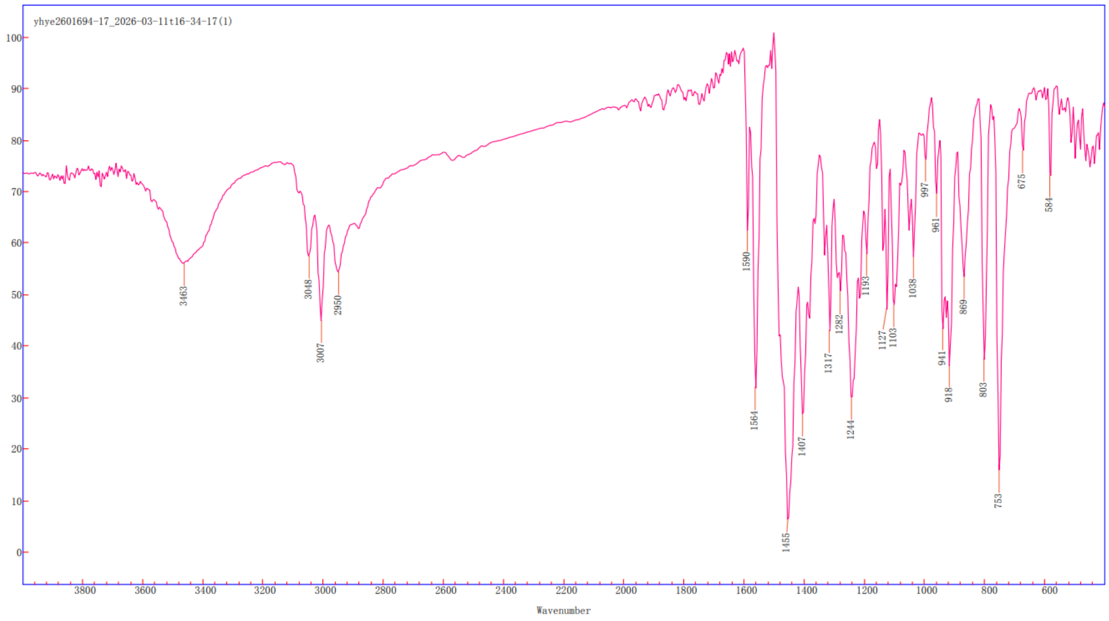

Figure S2-4 FT-IR of TMClPrAPtzCl.

Figure S3

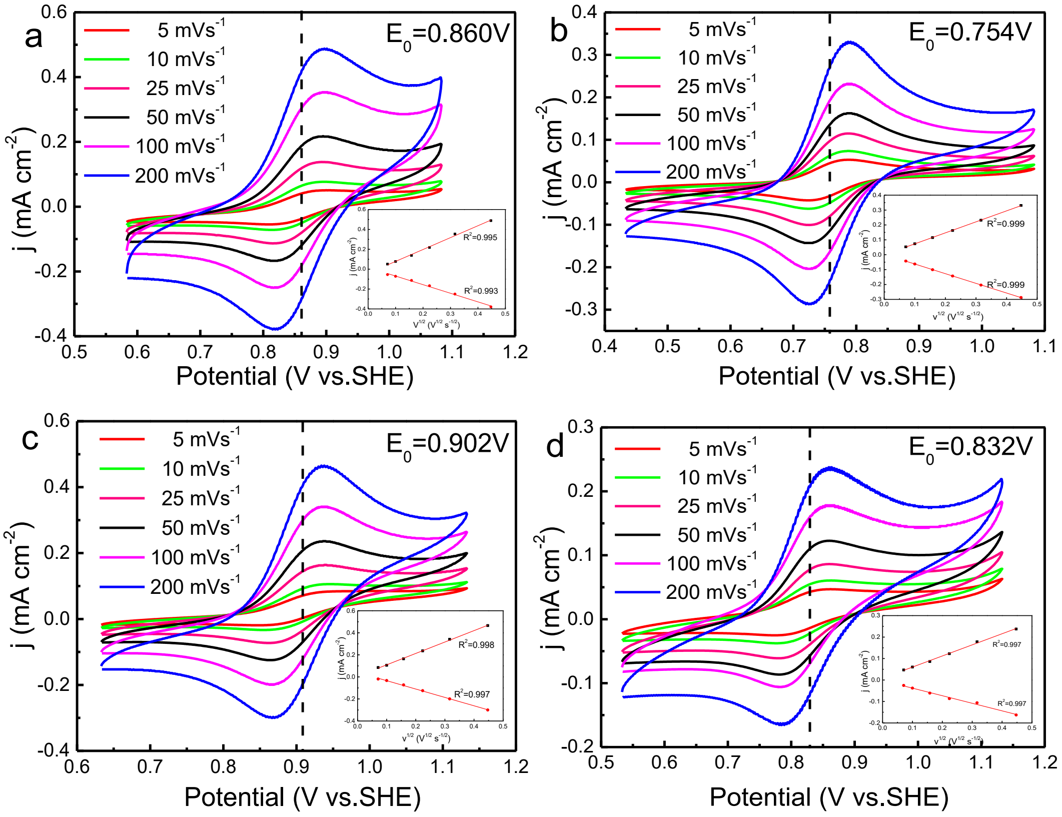

Figure S3 (a-d) CVs of 1 mM TMEtAPtzCl(a), TMPrAPtzCl(b), TMiPrAPtzCl(c) and TMClPrAPtzCl(d) in 1 M NaCl solution at scan rates of 5~200 mV s^{-1}. The inset is the relationship between the peak current densities (j_p) and the square root of the scan rate ($v^{1/2}$).

Figure S4

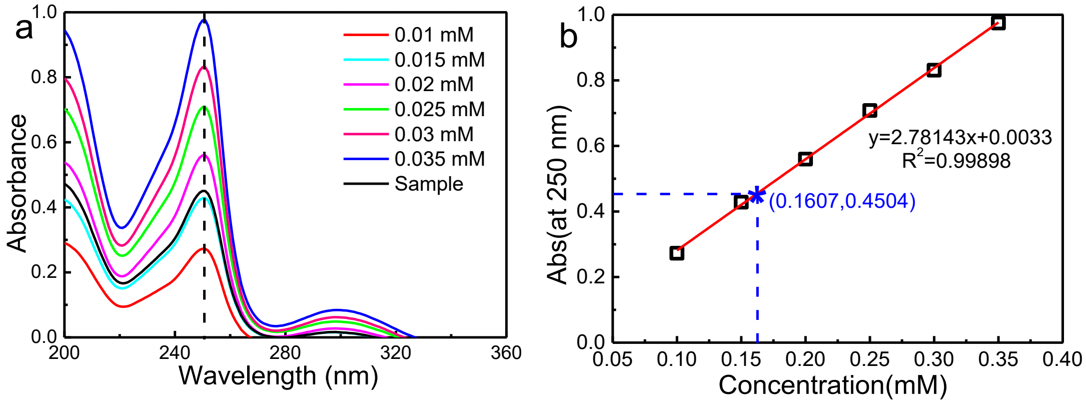

Figure S4-1 (a) UV-Vis absorption spectra of TMEtAPtzCl solutions with varying concentrations in deionized water. (b) Linear plot of TMEtAPtzCl concentration against

the maximum absorbance recorded at λ = 250 nm (10000-fold dilution). The solubility of TMEtAPtzCl in deionized water was determined to ~>1.61 M at 25±1℃ (calculated based on 1 L of solvent).

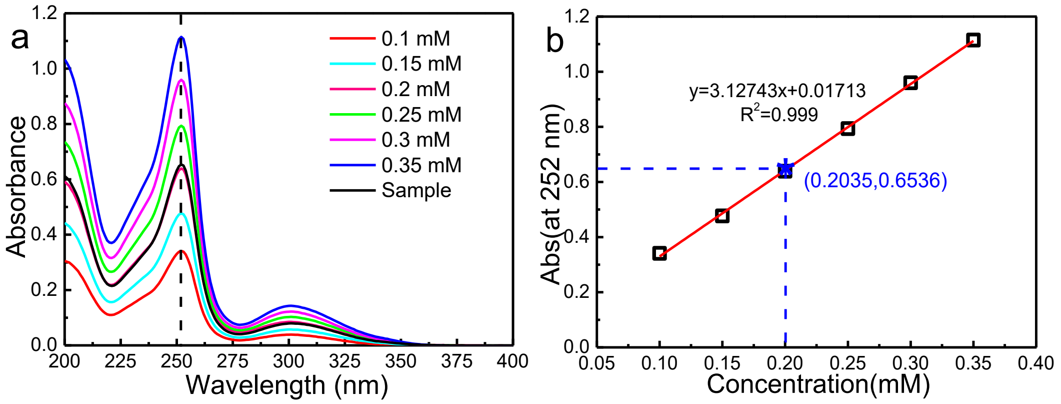

Figure S4-2 (a) UV-Vis absorption spectra of TMPrAPtzCl solutions with varying concentrations in deionized water. (b) Linear plot of TMPrAPtzCl concentration against the maximum absorbance recorded at λ = 252 nm (10000-fold dilution). The solubility of TMPrAPtzCl in deionized water was determined to be 2.03 M at 25±1℃ (calculated based on 1 L of solvent).

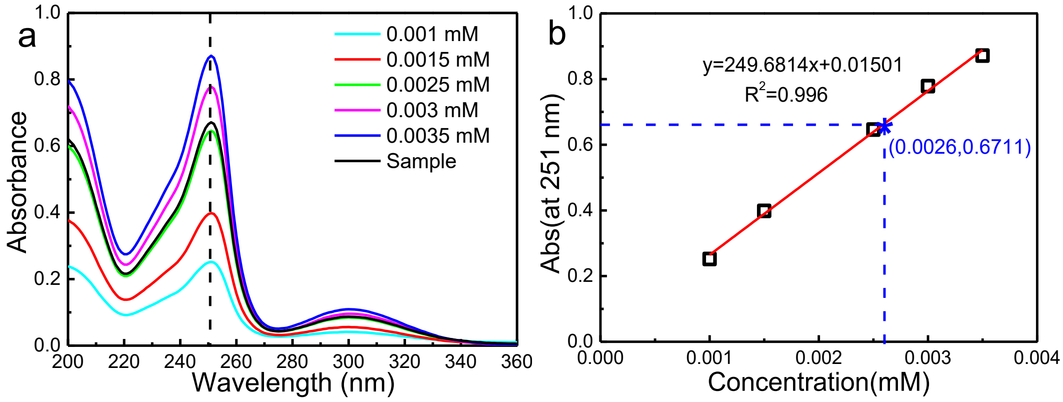

Figure S4-3 (a) UV-Vis absorption spectra of TMiPrAPtzCl solutions with varying concentrations in deionized water. (b) Linear plot of TMiPrAPtzCl concentration against the maximum absorbance recorded at λ = 251 nm (10000-fold dilution). The solubility of TMiPrAPtzCl in deionized water was determined to be 2.69 M at 25±1℃

(calculated based on 1 L of solvent).

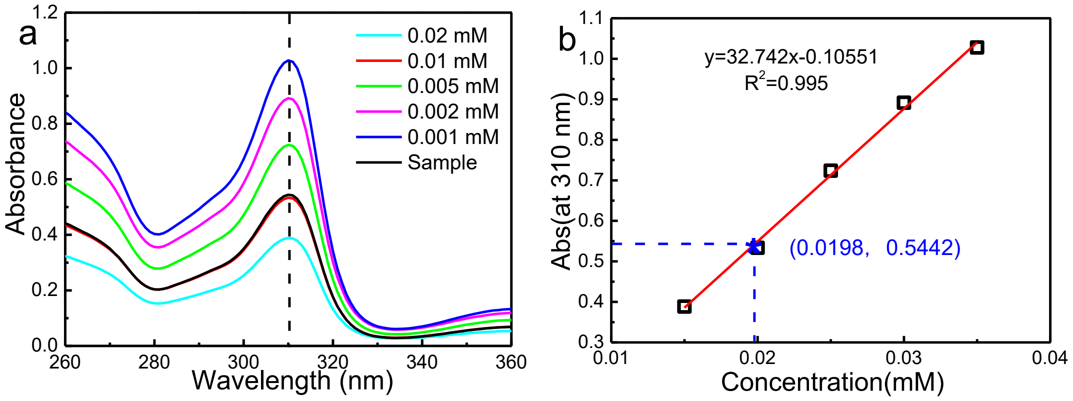

Figure S4-4 (a) UV-Vis absorption spectra of TMClPrAPtzCl solutions with varying concentrations in deionized water. (b) Linear plot of TMClPrAPtzCl concentration against the maximum absorbance recorded at λ = 310 nm (10000-fold dilution). The solubility of TMClPrAPtzCl in deionized water was determined to be 1.90 M at 25±1℃ (calculated based on 1 L of solvent).
